# Influence of repeated measurements on small bowel length

**DOI:** 10.1186/s40064-016-3557-7

**Published:** 2016-10-21

**Authors:** Servet Karagul, Cuneyt Kayaalp, Serdar Kirmizi, Ali Tardu, Ismail Ertugrul, Kerem Tolan, Fatih Sumer

**Affiliations:** Department of Surgery, Faculty of Medicine, Inonu University, Malatya, Turkey

**Keywords:** Anatomy, Bariatric surgery, Metabolic surgery, Short bowel syndrome, Small intestine

## Abstract

**Purpose:**

Measurement of small bowel length (SBL) is a common procedure in gastrointestinal surgery. When required, repeated SBL measurements can be done during surgery. Our aim was to evaluate whether these repeated measurements differ in SBL results.

**Methods:**

Small bowel length was measured during laparotomy in 28 patients between ligament of Treitz and caecum, using a standard measure, two times in each patient consecutively by two different surgeons from the anti-mesenteric border of the bowel.

**Results:**

The median age was 33 (19–67) including 18 male. There were 16 healthy donors for living related liver transplantations. Second measurements, performed immediately after the first measurements, significantly shortened the measured SBLs in the same patients (580 ± 103 vs. 485 ± 78 cm, p < 0.001).

**Conclusions:**

During surgery, repeated length measurements caused contractions in the small bowel and this resulted to a significant decrease in the SBL. This should be keep in mind to prevent mismeasurements.

## Background

Small bowel length (SBL) is an important issue in surgical practice, especially when performing small bowel resections, anastomosis or creating a small bowel stoma. It is also important in bariatric and metabolic surgery, short bowel syndrome and in small bowel transplantation (Hamoui et al. 2008; Scolapio 2004). Early studies on SBL measurements were done in cadavers (ex vivo) (Bryant 1924; Treves 1885; Underhill 1955). Subsequently in in vivo measurements, SBLs were found to be shorter than in cadavers, which was due to the tonus of the intestinal smooth muscles in living humans. Hosseinpour and Behdad, compared cadaveric SBLs with the lengths of the patients’ intra-operative SBLs and the mean values were found to be 632 and 460 cm, respectively. There were several studies on measuring the SBL length and the measurement methods (Backman and Hallberg 1974; Chiba and Boles 1984; Glehen et al. 2003; Guzman et al. 1977; Hillenbrand et al. 2015; Hosseinpour and Behdad 2008; Hounnou et al. 2002; Nordgren et al. 1997; Raines et al. 2015; Tacchino 2015; Teitelbaum et al. 2013). But no study yet focused on the influence of repeated measurements on the outcomes. The purpose of our study was to compare, the SBL results after consecutive two measurements in the same patients.

## Methods

Ethical committee approval was granted by Inonu University (114/2015) and the study was registered to clinicaltrials.gov (NCT02560064). Informed consent was given by all patients involved.

### Inclusion and exclusion criteria

Small bowel length was measured in patients over 18-year-old during open, elective abdominal surgery under general anaesthesia. Patients with previous abdominal surgery, peritonitis, intestinal obstruction, emergency surgery, intra-abdominal adhesions, inflammatory bowel disease, history of an intra-abdominal infection and an ongoing pregnancy were excluded.

### Measurement method

**Fig. 1:**
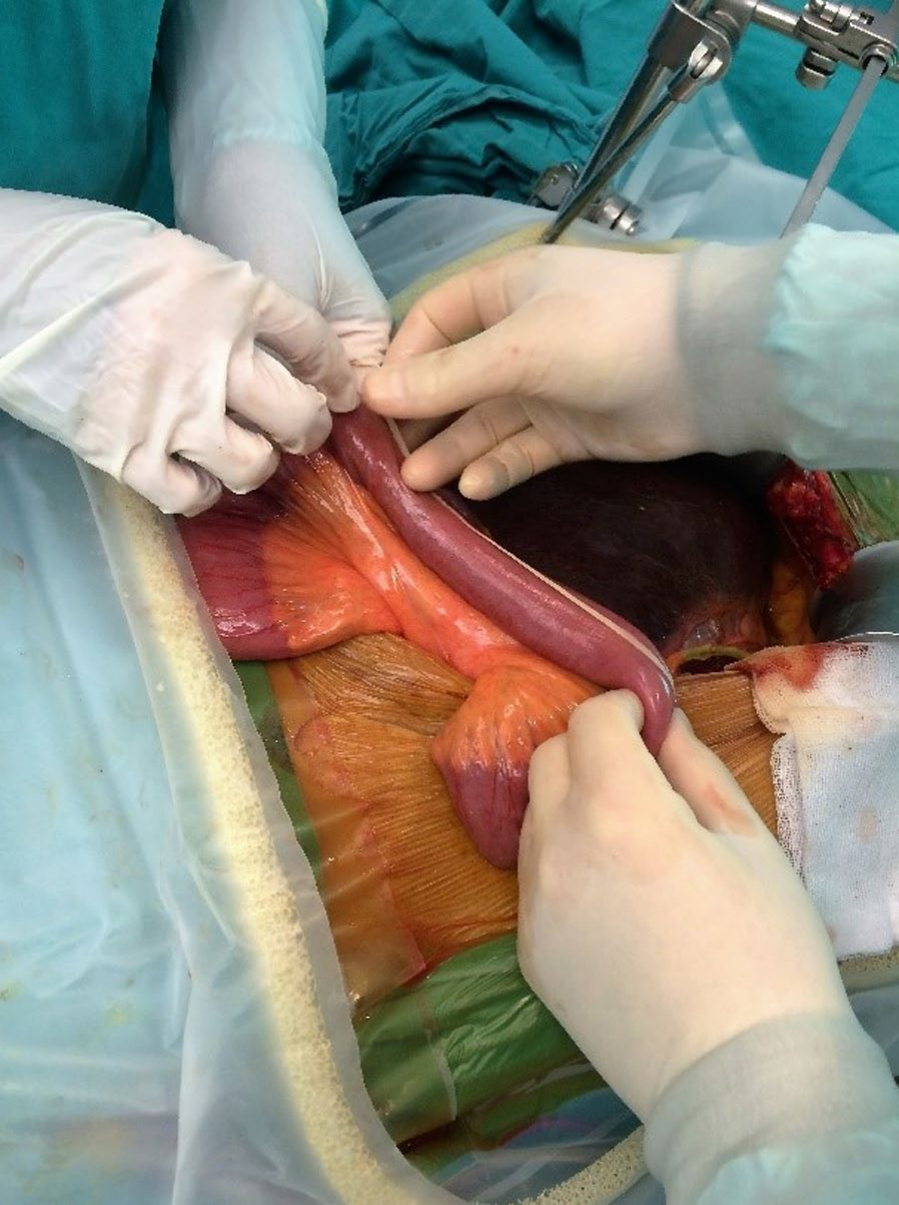
Small bowel measurement from anti-mesenteric border

Small bowel length between the ligament of Treitz and the ileo-caecal valve was measured. A standard 70 cm nylon tape was used as a measure. If the last segment of the bowel that was shorter than 70 cm, the remaining SBL was measured with the help of a ruler. The measurements were done from the anti-mesenteric border. This was done twice, by two of five surgeons. The selection of these two surgeons were not randomized and it was based on their surgical schedule in the operating room. Each these five surgeons did total 10–12 measurements as the first or the second measurements (Fig. 1). The first surgeon performed the measurement and second surgeon assisted for an appropriate measure. Immediately after the first measurement, the first surgeon replaced with the second one and assisted the second one in the same way. Alignment of the bowel was achieved by not letting the bowel fold onto itself, and care was taken not to stretch the bowel during measurements.

### Outcomes

In all patients, two measurements of the small bowel were done safely and successfully. Demographic data (gender, age, height, weight, body mass index) and the SBLs were recorded into a Microsoft Excel file.

### Statistics

If there was a homogenous distribution of the series, we used the paired student *t* test for comparison of the continuous variables of the same patients. If there was a non-homogenous distribution, we used the Mann–Whitney U-test. Comparison of two continuous parameters were done by Pearson correlation coefficient. SPSS 17.1 was used for the analysis. p < 0.05 was accepted as significant.

## Results

The mean age was 35.4 ± 13.0 (median and range 33 and 19–67) and 18 were male (64.3 %). The mean height was 169 ± 12 cm (median and range 172 and 147–186 cm), weight was 71.5 ± 14.2 kg (median and range 71.5 and 47–110 kg) and BMI was 25.1 ± 4.2 kg/m^2^ (median and range 24.8 and 18.6–37.8 kg/m^2^). Sixteen (57.1 %) of these patients were completely healthy subjects who were living donors for liver transplantation. Ten (35.5 %) had cirrhosis and the remaining two patients (7.1 %) were operated on for liver diseases not related to the small bowels (intrahepatic cholangiocarcinoma and liver hydatid disease). The time spent for two measurements was around 10 min for each patient (5 min for each measurement). There were no complications besides the prolongation of surgical duration.

**Fig. 2:**
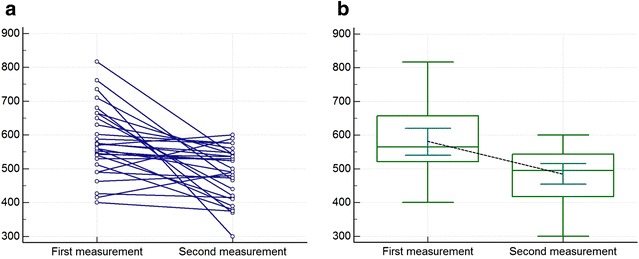
**a** Graphic of the first and second measurements. **b** Mean and standard deviation of the first and second measurements

A total of 56 SBL measurements were performed in 28 patients. It was observed that with repeated measurements, the measured SBLs got shorter (Fig. 2a, b). The first and the second measurements in the same patients were 580 ± 103 vs. 485 ± 78 cm, respectively (p < 0.001). The median and the range of the first and the second measurements were 565 cm (400–817 cm) and 495 cm (300–600 cm), respectively. There was a mean 95 cm shortening in the second measurements. The ratio of shortening was mean 14 %. The shortening ratio was not related with the diagnosis, age, gender, height, weight, or body mass index (Table 1).

**Table 1:** Degree of the shortening related with the patient parameters

Parameter^a^	Ratio of shortening (%)	p
Diagnosis		
Healthy subject (n: 16)	15.7 + 20.2	0.59
Liver disease (n: 12)	11.7 + 18.0	
Gender		
Male (n: 18)	11.3 + 21.4	0.59
Female (n: 10)	15.4 + 18.1	

Parameter^b^	Pearson correlation	p
Age	−0.27	0.16
Height	0.13	0.51
Weight	−0.06	0.76
Body mass index	−0.20	0.32

^a^Student-*t* test

^b^Pearson correlation coefficient

## Discussion

Measurement of SBL is a very common practice in abdominal surgery and sometimes surgeons do repeated SBL measurements to be clearer on the exact bowel distances before an anastomosis, resection or creating a stoma. However, this prudent attitude can cause some involuntary mismeasurements. We have demonstrated for the first time that the measured SBL is dramatically shortened after a repeated measurement, with this shortening reaching up to 14 %. We observed that there was obvious contractions of the small bowels after the first measurement and this resulted to a shorter measurements in the second.

Determining the small bowel dimensions is difficult compared to the other abdominal organs. Up to now, there exists no standardized SBL measurement technique used in the literature and we actually do not know the ideal measurement method. In living humans, a couple of different small bowel measurement techniques have been proposed (Hirsch et al. 1956; Shatari et al. 2004; Sinha et al. 2014). Several factors during SBL measurement can influence the results. Starting and ending reference points of the small bowel (including or excluding duodenum), measurement site at the small bowel (mesenteric, anti-mesenteric, midline), stretched or not stretched measurement will significantly influence the outcome.

Especially in bariatric-metabolic surgery, most of the surgeons use standard distances for the small bowel configurations (Abellan et al. 2014; Savassi-Rocha et al. 2008; Stefanidis et al. 2011). However they rarely measure the full SBL. In a study conducted among 211 American Society for Bariatric Surgery members who were performing laparoscopic gastric bypass surgery, it was shown that only four of them were measuring the length of the three limbs of small bowels during surgery (Madan et al. 2008). Looking at the bariatric-metabolic surgery context in particular, on the one hand there is the risk of short bowel syndrome and the risk of malnutrition; on the other hand there is the risk of weight regain and the continuation of co-morbidities. Most bariatric-metabolic surgeries are performed by laparoscopy and there is no study comparing SBL measurements by laparoscopy and open surgery. And some interesting experiences have been reported on the contradiction of the previous laparoscopic surgery and the later laparotomy measurements of the SBLs (Tacchino 2015).

Here, we clearly demonstrated that during surgical manipulations of small bowel, temporary and forceful contractions of the bowel can lead the surgeon to make involuntary mismeasurement at the SBL measurement. However, the questions that could not be answered here include the followings: How long will it take to return to the first SBL measurement? Is there any measurement technique that can obtain the same outcomes in the consecutive SBL measurements? In addition, this study had a limitation of including small number of the subjects. However, despite the limited numbers, we reached to a significant differences between the first and the second measurements and this provided us to make a clear comment.

Our centre has a Liver Transplantation Institute and at least half of our measurements were obtained from healthy donors for living-related liver transplantations. This study is, as far as we know, the first SBL measurement study performed in healthy subjects. Our ongoing projects will include comparing the measured SBLs of healthy and cirrhotic patients, demographic factors on SBL in completely healthy people, correlation between the liver volume and SBL, and comparing laparoscopic and open SBL measurements. The present study is the first step to finding a standardized SBL measurement method that can be used for all of the above studies.

## Conclusions

Repeated measurements cause contractions in the small bowel. Surgeons should keep in mind that there will be a significant decrease in the SBL when they perform a repeated measurement.
